# Rubisco packaging and stoichiometric composition of the native β-carboxysome in *Synechococcus elongatus* PCC7942

**DOI:** 10.1093/plphys/kiae665

**Published:** 2024-12-16

**Authors:** Yaqi Sun, Yuewen Sheng, Tao Ni, Xingwu Ge, Joscelyn Sarsby, Philip J Brownridge, Kang Li, Nathan Hardenbrook, Gregory F Dykes, Nichola Rockliffe, Claire E Eyers, Peijun Zhang, Lu-Ning Liu

**Affiliations:** Department of Biochemistry, Cell and Systems Biology, Institute of Systems, Molecular and Integrative Biology, University of Liverpool, Crown Street, Liverpool L69 7ZB, UK; Diamond Light Source, Harwell Science and Innovation Campus, Didcot OX11 0DE, UK; Division of Structural Biology, Wellcome Trust Centre for Human Genetics, University of Oxford, Oxford OX3 7BN, UK; Department of Biochemistry, Cell and Systems Biology, Institute of Systems, Molecular and Integrative Biology, University of Liverpool, Crown Street, Liverpool L69 7ZB, UK; Centre for Proteome Research, Institute of Systems, Molecular and Integrative Biology, University of Liverpool, Crown Street, Liverpool L69 7ZB, UK; Centre for Proteome Research, Institute of Systems, Molecular and Integrative Biology, University of Liverpool, Crown Street, Liverpool L69 7ZB, UK; College of Marine Life Sciences, and Frontiers Science Center for Deep Ocean Multispheres and Earth System, Ocean University of China, Qingdao 266003, China; Division of Structural Biology, Wellcome Trust Centre for Human Genetics, University of Oxford, Oxford OX3 7BN, UK; Department of Biochemistry, Cell and Systems Biology, Institute of Systems, Molecular and Integrative Biology, University of Liverpool, Crown Street, Liverpool L69 7ZB, UK; Faculty of Health & Life Sciences, GeneMill, University of Liverpool, Crown Street, Liverpool L69 7ZB, UK; Centre for Proteome Research, Institute of Systems, Molecular and Integrative Biology, University of Liverpool, Crown Street, Liverpool L69 7ZB, UK; Diamond Light Source, Harwell Science and Innovation Campus, Didcot OX11 0DE, UK; Division of Structural Biology, Wellcome Trust Centre for Human Genetics, University of Oxford, Oxford OX3 7BN, UK; Chinese Academy of Medical Sciences Oxford Institute, University of Oxford, Oxford OX3 7BN, UK; Department of Biochemistry, Cell and Systems Biology, Institute of Systems, Molecular and Integrative Biology, University of Liverpool, Crown Street, Liverpool L69 7ZB, UK; College of Marine Life Sciences, and Frontiers Science Center for Deep Ocean Multispheres and Earth System, Ocean University of China, Qingdao 266003, China

## Abstract

Carboxysomes are anabolic bacterial microcompartments that play an essential role in CO_2_ fixation in cyanobacteria. This self-assembling proteinaceous organelle uses a polyhedral shell constructed by hundreds of shell protein paralogs to encapsulate the key CO_2_-fixing enzymes Rubisco and carbonic anhydrase. Deciphering the precise arrangement and structural organization of Rubisco enzymes within carboxysomes is crucial for understanding carboxysome formation and overall functionality. Here, we employed cryoelectron tomography and subtomogram averaging to delineate the 3D packaging of Rubiscos within β-carboxysomes in the freshwater cyanobacterium *Synechococcus elongatus* PCC7942 grown under low light. Our results revealed that Rubiscos are arranged in multiple concentric layers parallel to the shell within the β-carboxysome lumen. We also detected Rubisco binding with the scaffolding protein CcmM in β-carboxysomes, which is instrumental for Rubisco encapsulation and β-carboxysome assembly. Using Quantification conCATamer-based quantitative MS, we determined the absolute stoichiometric composition of the entire β-carboxysome. This study provides insights into the assembly principles and structural variation of β-carboxysomes, which will aid in the rational design and repurposing of carboxysome nanostructures for diverse bioengineering applications.

## Teaser

Cryoelectron tomography and absolute quantitative MS reveal Rubisco organization and stoichiometric composition of native β-carboxysomes, shedding light on carboxysome structure.

## Introduction

Biological CO_2_ fixation is an essential process in living organisms that enables the conversion of inorganic CO_2_ into organic compounds, supporting the global carbon cycle and sustaining life on Earth. The key CO_2_-fixation enzyme, ribulose-1,5-bisphosphate carboxylase/oxygenase (Rubisco), captures and converts inorganic carbon to produce a sugar precursor through the Calvin–Benson–Bassham cycle. However, Rubisco is surprisingly inefficient, making its catalytical reactions the limiting steps in photosynthetic CO_2_ fixation. The ineffectiveness of Rubisco originates from its slow catalytic rate and poor capability in discriminating between CO_2_ and O_2_. To overcome the inherent limitations, distinct organisms, including many bacteria, algae, C_4_ plants, and crassulacean acid metabolism plants, have evolved various CO_2_-concentrating mechanisms (CCMs) to accumulate CO_2_ around Rubisco (Field et al. 1998; McGrath and Long 2014; Gonzalez-Esquer et al. 2016; Long et al. 2016; Rae et al. 2017). In contrast, an overwhelming majority of agricultural crops, namely C_3_ plants, lack any form of CCM, resulting in a lower photosynthetic efficiency (Price et al. 2013). Deciphering how the CCM systems work in nature and introducing a functional CCM into crop plants to supercharge CO_2_ fixation in C_3_ plants and enhance crop yields has received increasing attention.

**Figure 1.**
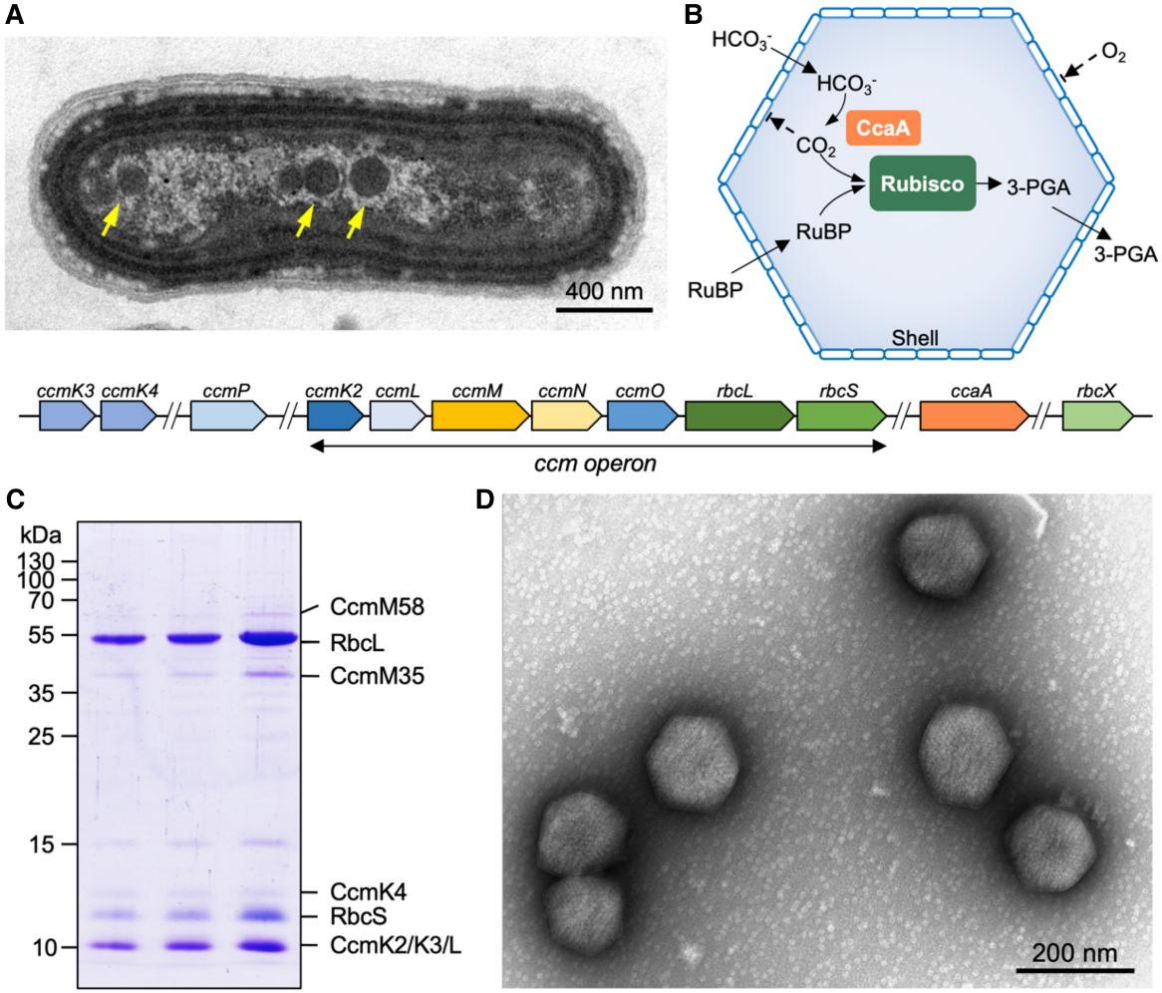
Purification and characterization of Syn7942 β-carboxysomes. **A)** Thin-section electron microscopy of a Syn7942 cell with β-carboxysomes indicated by arrows (top) and the organization of genes expressing β-carboxysome proteins in the Syn7942 genome (bottom), **B)** Functional diagrams of β-carboxysomes. The carboxysome shell is selectively permeable, allowing HCO_3_^−^ and RuBP to enter while blocking O_2_ influx and preventing CO_2_ leakage to the cytoplasm. Within the β-carboxysome lumen, CcaA catalyzes the dehydration of HCO_3_^−^ to CO_2_, providing elevated levels of CO_2_ around the Rubisco active sites in the carboxysome lumen to enhance CO_2_ fixation, **C)** SDS–PAGE of purified β-carboxysomes from 3 biological replicates. The bands near 15 kDa likely corresponded to egg lysozyme (14.4 kDa) used during purification, as determined previously (Long et al. 2005; Faulkner et al. 2017), and **D)** electron microscopy image of isolated β-carboxysomes from Syn7942.

Carboxysomes are a family of bacterial microcompartments responsible for CO_2_ fixation and serve as the central component of CCMs in cyanobacteria and some proteobacteria (Kerfeld and Erbilgin 2015; Liu 2022). The carboxysome sequesters the key CO_2_-fixing enzyme, ribulose 1,5-bisphosphate carboxylase/oxygenase (Rubisco), and carbonic anhydrase (CA), using a polyhedral protein shell (Fig. 1A). The carboxysome shell is constructed from multiple protein paralogs (including hexamers, pentamers, and trimers) through self-assembly. Their intrinsic architectural properties enable carboxysomes to play a crucial role in the global carbon cycle and primary productivity (Behrenfeld et al. 2001; Rae et al. 2013; Faulkner et al. 2017; Kerfeld et al. 2018; Sun et al. 2019; Sun et al. 2022). Understanding the assembly and organizational principles of carboxysomes is of key importance not only for elucidating carboxysome function and catalytic performance but also for harnessing their potential in synthetic engineering for various biotechnological applications (Liu 2021; Liu et al. 2021).

There are 2 carboxysome lineages, α-carboxysomes and β-carboxysomes, which differ in the types of encased Rubisco, the composition of building proteins, subcellular distribution, and biogenesis pathway (Price et al. 2008; Rae et al. 2013; Kerfeld and Melnicki 2016). For example, α-carboxysomes encapsulate Form 1A Rubisco, and their biogenesis may undertake simultaneous and/or independent Rubisco core assembly and shell encapsulation (Iancu et al. 2010); in contrast, β-carboxysomes encapsulate Form 1B Rubisco, exhibit even distribution inside the cells (Savage et al. 2010), and de novo assembly of β-carboxysomes exploits the “inside–out” model (Cameron et al. 2013; Chen et al. 2013). Despite the evolutionary and structural differences, it has been indicated that the diverse Rubisco forms found in α- and β-carboxysomes exhibited similar kinetic parameters, and both types of carboxysomes may possess comparable functional capabilities (Whitehead et al. 2014).

The β-carboxysome of the cyanobacterium *Synechococcus elongatus* PCC7942 (Syn7942) has been extensively characterized as a model carboxysome (Fig. 1B). The cargo enzymes of the Syn7942 β-carboxysome include Form IB Rubisco (comprising Rubisco large and small subunits RbcL and RbcS), which have a higher catalytic rate than that of plant Rubisco (Whitney et al. 2011; Orr et al. 2020), as well as β-CA (also known as CcaA). The shell consists of a series of proteins encoded by the *ccm* operon, including the structural proteins CcmK2, CcmK3, and CcmK4, which appear as hexamers and predominantly form shell facets (Kerfeld et al. 2005), CcmL pentamers that occupy the vertices of the polyhedron (Tanaka et al. 2008), as well as the trimeric proteins CcmO and CcmP (Cai et al. 2013; Larsson et al. 2017). CcmM presents in 2 isoforms, CcmM35 and CcmM58, both of which are essential for β-carboxysome assembly (Long et al. 2007, 2010). While both CcmM35 and CcmM58 act as “linkers” to promote Rubisco nucleation in β-carboxysome biogenesis, CcmM58 binds with CcmN to facilitate shell-interior association (Eisenhut et al. 2007; Long et al. 2007, 2010; Kinney et al. 2012; Gonzalez-Esquer et al. 2015; Ryan et al. 2019; Wang et al. 2019), essential for de novo assembly of β-carboxysomes (Cameron et al. 2013; Chen et al. 2013). Moreover, auxiliary factors Raf1 and RbcX are involved in β-carboxysome formation and regulation. Raf1 mediates the assembly of Rubisco holoenzymes and the biogenesis of intact β-carboxysomes (Huang et al. 2020). RbcX serves as a component of the carboxysome and plays a role in mediating β-carboxysome assembly and subcellular distribution (Huang et al. 2019). In the β-carboxysomes that contain CcmK1, a chaperon protein CcmS has recently been determined to play an important role in mediating β-carboxysome formation through interaction with the CcmK1 C-termini exposing at the outer surface of the shell (Chen et al. 2023; Cheng et al. 2024).

Despite the substantial efforts made over the past decades to understand the functions of individual carboxysome components, the precise composition of carboxysomes and how the building proteins assemble and self-organize to form intact carboxysome structures remain fundamental questions. Recent studies have provided detailed information on the encapsulation and spatial arrangement of Rubiscos within α-carboxysomes (Metskas et al. 2022; Ni et al. 2022a, 2022b; Evans et al. 2023; Ni et al. 2023), as well as the exact protein stoichiometry of α-carboxysomes (Sun et al. 2022). The β-carboxysomes exhibit structural and compositional variations under different environmental conditions; for example, β-carboxysomes can vary in size, with diameters spanning from 144 to 208 nm, depending on the light and CO_2_ conditions (Sun et al. 2019). A recent study characterized the Rubisco organization of β-carboxysomes isolated from Syn7942, which have a diameter of 193 nm (Kong et al. 2024). However, how the Rubisco organization is modulated within the β-carboxysomes of varying sizes, as well as the precise composition of intact β-carboxysomes, remains unclear. This knowledge gap is among the major obstacles in the construction of functional intact β-carboxysomes in nonnative organisms for bioengineering applications.

In this study, we isolated intact β-carboxysomes with a reduced diameter of 169 nm from Syn7942 cells that were cultivated under low-light conditions and determined the spatial packaging of Rubisco mediated by CcmM within the smaller β-carboxysome using cryoelectron tomography (cryoET). Moreover, we performed absolute quantification of protein components within the native β-carboxysome using Quantification conCATamer (QconCAT)-assisted quantitative MS in combination with biochemical analysis and enzymatic assays. Our results provide a structural basis for a detailed understanding of the assembly principles and structural regulation of β-carboxysomes. Advanced knowledge of carboxysome assembly and modulation will aid in the rational design and synthetic engineering of carboxysome-based structures.

## Results

### Structure and assembly of Rubisco in isolated Syn7942 β-carboxysomes

Our previous studies indicated that β-carboxysomes produced under low-light conditions tend to be smaller in diameter and more uniform in shape (Sun et al. 2019), which may make them suitable for in-depth structural and biochemical analysis. Therefore, we purified intact β-carboxysomes from Syn7942 grown under a naturally relevant lower light (15 μE m^−2^ s^−1^) using sucrose gradient ultracentrifugation (Faulkner et al. 2017) (Supplementary Fig. S1A). The β-carboxysome proteins were identified by SDS-polyacrylamide gel electrophoresis (SDS–PAGE) (Fig. 1C) and immunoblot analysis (Supplementary Fig. S1B). Negative-staining electron microscopy (EM) revealed that the isolated β-carboxysomes formed intact polyhedral shapes with clear borders (Fig. 1D). The CO_2_-fixation rate of isolated β-carboxysomes was measured as 2.56 ± 0.16 *μ*mol mg^−1^ min^−1^ (*n* = 3 independent biological samples), which is comparable to that of α-carboxysomes from the chemoautotrophic bacterium *Halothiobacillus neapolitanus*, when tested under the same experimental conditions (Sun et al. 2022). This is consistent with previous findings (Whitehead et al. 2014). These results confirmed the functional and structural integrity of β-carboxysomes isolated from Syn7942.

**Figure 2.**
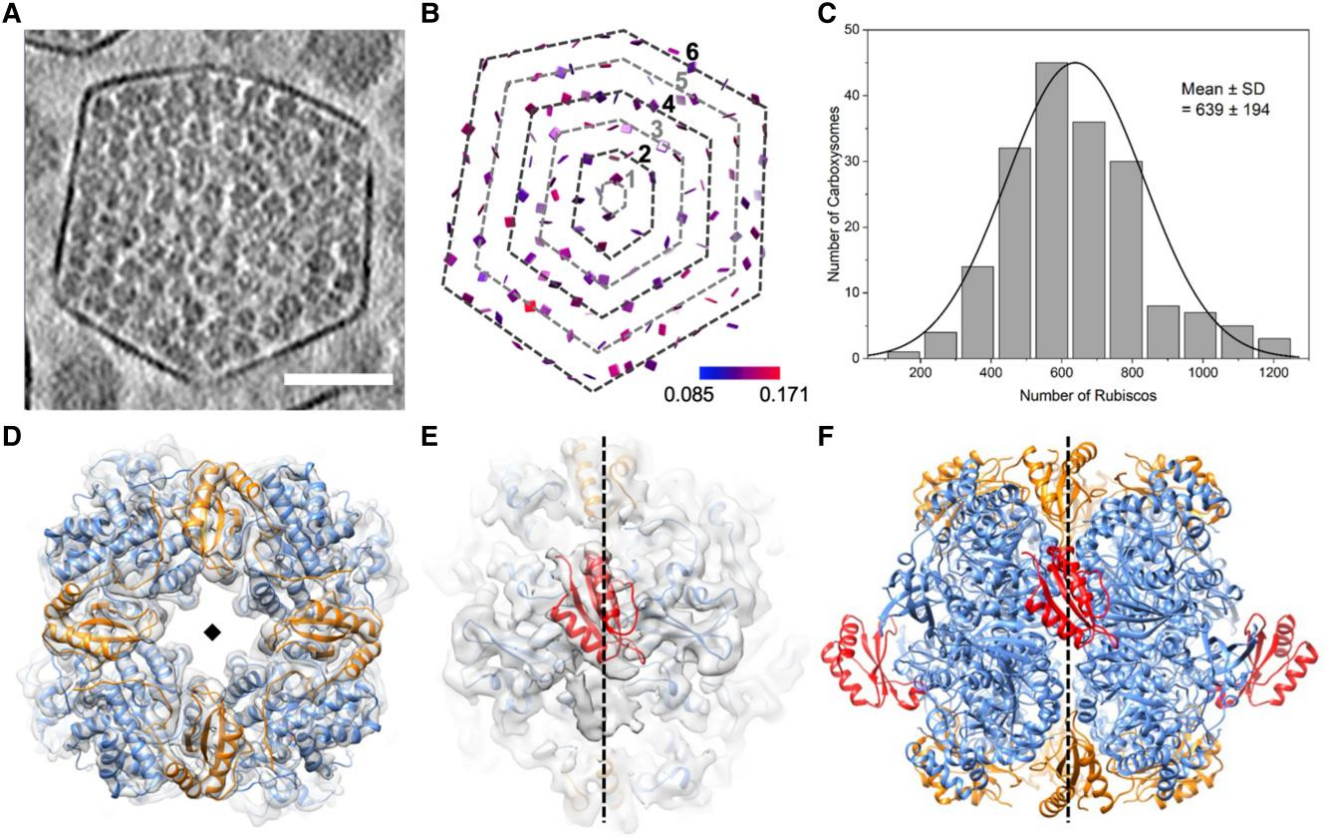
CryoEM structures and organization of Rubisco within β-carboxysomes. **A)** A tomogram slice showing a typical β-carboxysome. Scale bar: 50 nm. **B)** The position and orientation of individual Rubisco mapped back to the tomogram to the tomogram of β-carboxysome in **A)**, shown as a square plate perpendicular to the 4-fold symmetry axis of Rubisco and colored according to the cross-correlation values (0.085 to 0.171, blue to red) between individual Rubisco and the STA map. It contains a total of 6 layers and the layer number is defined from the core to the shell, as shown. **C)** Histogram of Rubisco numbers in β-carboxysomes (*n* = 185). **D)** The cryoET subtomogram averaged structures of Rubisco at 3.5 Å, overlapped with refined atomic model (PDB ID: 8BCM). RbcL subunits are shown in blue and RbcS subunits are shown in orange. **E)** Zoomed-in view showing the density of CcmM, the linker protein which binds Rubisco, fitted with the atomic model of CcmM SSUL (red, PDB ID: 6HBC). CcmM exhibits 2 binding modes (upper groove and lower groove, Supplementary Fig. S5). RbcL subunits are shown in blue and RbcS subunits are shown in orange. **F)** The overall atomic models of Rubisco along with 4 SSUL domains in 2 views. Only one binding mode is shown (upper groove). RbcL, RbcS, and CcmM are shown in blue, orange, and red, respectively. The diamond **(D)** and dashed black lines **(E**, **F)** indicate the 4-fold axis.

We performed cryoET and subtomogram averaging (STA) of the isolated β-carboxysomes using emClarity (Himes and Zhang 2018; Ni et al. 2022a, 2022b) (Fig. 2, Supplementary Table S1). The β-carboxysomes exhibited morphological variations with a size of 169.0 ± 11.8 nm (Supplementary Fig. S1C), which falls within the range of the diameters determined within and isolated from Syn7942 cells in previous studies (Faulkner et al. 2017; Sun et al. 2019). Individual Rubiscos inside the β-carboxysomes were readily delineated in the raw tomograms (Fig. 2A, Supplementary Fig. S2, Supplementary Video S1). Template matching and mapping of the position and orientation of individual Rubisco complexes to the original tomograms revealed that Rubiscos had a paracrystalline arrangement within the β-carboxysome lumen, forming 4 to 9 concentric layers (Fig. 2B, Supplementary Fig. S3A). Among the β-carboxysome particles analyzed, 92.4% had 4 to 6 layers of Rubiscos, with 5 layers being the most frequently observed (44.3%, Supplementary Fig. S3A). The pairwise distance between neighboring Rubiscos is 121.9 ± 31.4 Å (Supplementary Fig. S3B), which is comparable to that of α-carboxysomes (128 to 129 Å) (Ni et al. 2022a, 2022b). Our analysis reveals that each β-carboxysome contains ∼639 ± 194 Rubisco complexes (Fig. 2C). This quantity is remarkably greater than that of the α-carboxysomes from *Cyanobium* 7001, *H. neapolitanus*, and *Prochlorococcus* (Ni et al. 2022a, 2022b; Evans et al. 2023; Zhou et al. 2024). However, the Rubisco content is notably lower than that observed in larger β-carboxysomes with a diameter of 193 nm (Kong et al. 2024). Whether this variation in Rubisco content within distinct β-carboxysomes is attributed to differences in carboxysome diameter, the spacing between Rubisco pairs, or other factors merits further investigation.

As expected, the content of Rubisco per layer and total Rubiscos per β-carboxysome gradually increased as the number of layers increased from the core center to the shell (Supplementary Fig. S3, C and D). Approximately, layers 1 to 6 contain 1, 20, 71, 174, 308, and 407 copies of Rubiscos, respectively (Supplementary Fig. S3C). This leads to ∼600 copies of Rubiscos per Syn7942 β-carboxysome, given that the majority of β-carboxysomes contain 5 layers of Rubiscos (Supplementary Fig. S3A). The inner 4 layers are closely packed and relatively ordered; therefore, we only considered the data within the first 2 to 4 layers to establish a model/formula for the number of Rubiscos for each layer. The prediction was based on the radial distances of Rubiscos from the core as well as the sphere surface area formula (4*πr*^2^). The average distance between 2 Rubisco complexes is ∼120 Å (Supplementary Fig. S3B), as is the distance between 2 consecutive layers. When taking layer 4 as the reference to estimate the total number of Rubiscos for layers 2 and 3, we found that the estimated results closely matched the actual numbers (Supplementary Fig. S3C). Accordingly, this allowed us to extrapolate the number of Rubiscos for layers 5 to 9 in a β-carboxysome that has 9 layers (Supplementary Fig. S3D). Moreover, Rubiscos are mostly oriented with their 4-fold axis along the radical direction (90 °) across all layers (Supplementary Fig. S4, A to C). Interestingly, no significant difference in the orientation of Rubisco was observed within the outermost layer compared to the inner layers (Supplementary Fig. S4D). This suggests that the size of the shell/carboxysome does not have a significant effect on the internal Rubisco arrangement, in line with the “Core first” assembly pathway of β-carboxysomes (Cameron et al. 2013). In contrast, a recent study of β-carboxysomes proposed that the Rubiscos at the outermost layer of β-carboxysomes have a more regular arrangement (Kong et al. 2024). This discrepancy might be ascribed to the structural plasticity of the β-carboxysomes formed under different growth conditions (see detailed discussion below).

We further determined the structure of Rubisco within native Syn7942 β-carboxysomes at a resolution of 3.5 Å using STA (Fig. 2D, Supplementary Fig. S2, Supplementary Video S2). The resolved structure of the Rubisco L_8_S_8_ hexadecamer was oriented along its 4-fold axis along the radial direction, in good agreement with the reported Syn7942 Rubisco structures (Wang et al. 2019; Huang et al. 2020; Kong et al. 2024; Sun et al. 2024). Intriguingly, an extra density in the groove at the interface between antiparallel RbcL dimers was well-resolved (Fig. 2, E and F). This density matches the Rubisco small subunit-like (SSUL) module of the “linker” protein CcmM, which contains 2 α-helices packed against a β-sheet (Fig. 2E). As it sits on the 2-fold axis, the 2-fold symmetrized density appears a half intensity compared to Rubisco, and space can only accommodate 1 module. We thus deduced that one copy of CcmM SSUL can bind to the RbcL groove in either the up or down configuration, but not both (Fig. 2E, Supplementary Fig. S5, Supplementary Video S3). The binding of CcmM SSUL to Rubisco is reminiscent of that of the reconstituted Rubisco–CcmM35 complex [Protein Data Bank (PDB) ID: 6HBC] (Wang et al. 2019). Each Rubisco within the carboxysome has potentially 4 CcmM SSUL domains that can bind Rubisco at the RbcL dimer groove in 2 different modes (Fig. 2, E and F). These cryoET results provide direct evidence of Rubisco–CcmM interactions within β-carboxysomes, which drive the recruitment and packaging of Rubisco to form paracrystalline arrays in the β-carboxysome lumen.

### In situ cryoET of β-carboxysomes

**Figure 3.**
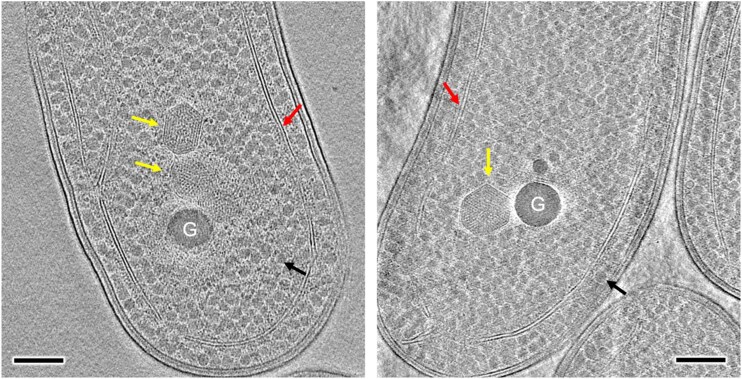
In situ cryoET of frozen-hydrated Syn7942 cell lamella. Yellow arrows indicate the β-carboxysome structures which possess regularly packed Rubisco in the β-carboxysome lumen. G represents large electron-dense polyphosphate granule. Red arrows indicate thylakoid membranes and black arrows indicate glycogen granules. Scale bar, 200 nm. See also Supplementary Video S4.

We further conducted in situ cryoET to explore the structure of β-carboxysomes in native Syn7942 cells (Fig. 3). The intact β-carboxysome particles exhibit polyhedral shapes and a concentric ring-like arrangement of Rubisco within the carboxysome lumen, corroborating our observations from purified β-carboxysomes (Fig. 2). The observed in vivo structural variability resembles that observed in previous studies of Syn7942 and the findings of α-carboxysomes (Sun et al. 2022). Additionally, β-carboxysomes were located away from the edge of the cell, often accompanied by large electron-dense granules in proximity (Fig. 3, Supplementary Video S4). This likely suggests the physical connections between β-carboxysomes and granules, in line with the observations for α-carboxysomes in *H. neapolitanus* cells (Iancu et al. 2010).

### Protein stoichiometry of β-carboxysomes

**Figure 4.**
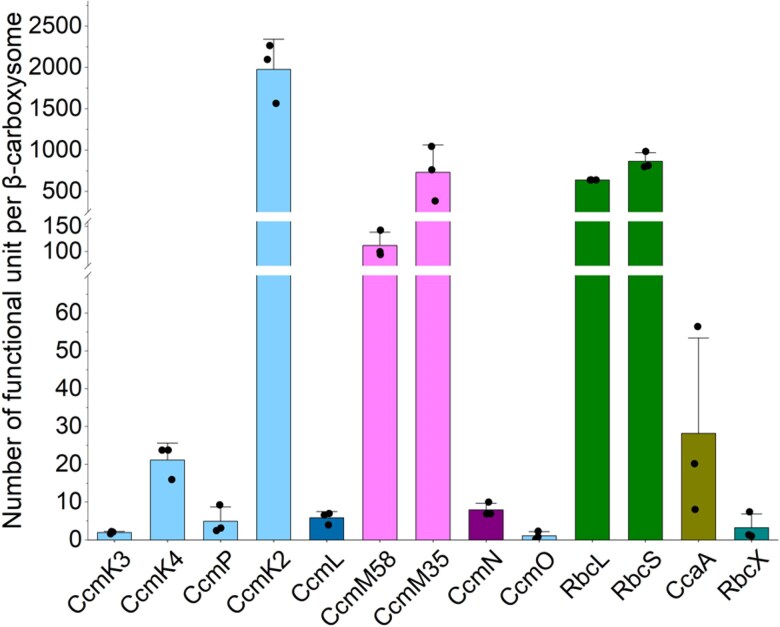
Absolute quantification of the subunit stoichiometry of Syn7942 β-carboxysomes using QconCAT standardization. See also the content of protein oligomers per β-carboxysome in Table 1. Data are presented as means ± Sd from 4 independent biological replicates.

Owing to the irregular morphology of Syn7942 β-carboxysomes, recognition of additional crucial carboxysome components aside from Rubiscos remains unfeasible via the cryoET approach. Using fluorescence tagging and single-molecule fluorescence microscopy, our previous work has estimated the protein abundance of β-carboxysomes in vivo (Sun et al. 2019). However, given the potential effects of fluorescence tagging and limitations of imaging sensitivity, the precise protein stoichiometry of the β-carboxysome has not been determined. By employing MS-based absolute multiplexed protein quantification using a labeled QconCAT (Rivers et al. 2007; Simpson and Beynon 2012; Johnson et al. 2021), we previously decoded the accurate stoichiometric composition of protein components of α-carboxysomes and Pdu metabolosomes (Yang et al. 2020; Sun et al. 2022). To establish the stoichiometry of the β-carboxysome components, we employed high-resolution liquid chromatography-MS (LC-MS) with protein-specific stable isotope-labeled internal standards created using the QconCAT approach. This allowed us to determine the absolute stoichiometry of all the protein components in the purified Syn7942 β-carboxysomes (Fig. 4, Table 1). Thus, we provide precise quantification of the protein stoichiometry of the β-carboxysome.

**Table 1.** Protein stoichiometry of Syn7942 β-carboxysome determined by QconCAT

Category	Protein	Structure of functional unit	Molecular mass	% of total protein	Units of monomers per carboxysome	Functional units of multimer per carboxysome
Structural proteins	CcmK2	Hexamer (PDB: 4OX7) (Cai et al. 2015)	10.9 kDa	24.3 ± 2.6	11,853 ± 2191	1,975 ± 365
	CcmK3	Hexamer^a^	10.8 kDa	0.03 ± 0.01	12 ± 2	2 ± 0
	CcmK4	Hexamer (PDB: 4OX6) (Cai et al. 2015)	11.9 kDa	0.29 ± 0.05	127 ± 27	21 ± 4
	CcmL	Pentamer (PDB: 2QW7) (Tanaka et al. 2008)	11.0 kDa	0.07 ± 0.02	29 ± 8	6 ± 2
	CcmO	Pseudohexamer^a^	29.4 kDa	0.04 ± 0.05	7 ± 7	1 ± 1
	CcmP	Pseudohexamer (PDB: 5LSR) (Larsson et al. 2017)	23.2 kDa	0.14 ± 0.12	30 ± 22	5 ± 4
	CcmM35	Monomer	35.2 kDa	4.8 ± 1.8	730 ± 331	730 ± 331
	CcmM58	Monomer	57.8 kDa	1.22 ± 0.21	112 ± 27	112 ± 27
	CcmN	Monomer	16.3 kDa	0.03 ± 0.01	8 ± 2	8 ± 2
Catalytic proteins	RbcL	L_8_S_8_ hexadecamer (PDB: 1RBL) (Newman et al. 1993)	52.4 kDa	50.9 ± 4.9	5,112	639
	RbcS	L_8_S_8_ hexadecamer (PDB: 1RBL) (Newman et al. 1993)	13.3 kDa	17.4 ± 1	6,919 ± 825	865 ± 103
	RbcX	Dimer (PDB: 2PY8) (Tanaka et al. 2007)	17.0 kDa	0.03 ± 0.03	7 ± 7	3 ± 4
	CcaA	Hexamer (PDB: 5SWC) (McGurn et al. 2016)	30.2 kDa	0.93 ± 0.76	169 ± 151	28 ± 25
Intact β-carboxysome			529.4 MDa			

^a^Based on the sequence homology of CcmK3 and CcmK4, and of CcmO and CcmP. Data are presented as means ± Sd from 4 independent biological replicates.

The single QconCAT peptide was designed to contain 2 unique quantification peptides (Q-peptides) for each Syn7942 β-carboxysome protein: CcmK2, CcmK3, CcmK4, CcmL, the CcmM35/58 shared region, CcmM58, CcmN, CcmO, RbcL, RbcS, CcmP, RbcX, and CcaA (Supplementary Fig. S6A, Supplementary Tables S2 and S3). The stable isotope-labeled QconCAT was purified following peptide expression using a cell-free synthesis system (Takemori et al. 2017) (Supplementary Fig. S6B), prior to LC-MS-based absolute protein quantification. To determine the absolute amounts of the β-carboxysome protein components, we prepared fresh isolates and co-digested them with purified isotope-labelled QconCAT. All β-carboxysome proteins were successfully detected (Supplementary Data Set 1), enabling us to determine the abundance of protein components within a single β-carboxysome (Fig. 4, Table 1). This quantification was based on the Rubisco content determined for the β-carboxysome with an average diameter of 169.0 nm, as measured using cryoET (Supplementary Fig. S1C). Our results revealed that the most abundant proteins in the Syn7942 β-carboxysome were CcmK2 hexamers (1,975 copies), followed by Rubisco (639 copies, quantified based on the content of RbcL), CcmM35 (730 copies), CcmM58 (112 copies), β-CA (CcaA) hexamers (McGurn et al. 2016) (28 copies), CcmK4 hexamers (21 copies), CcmP pseudo-hexamers (5 copies), CcmN monomers (8 copies), CcmK3 (2 copies of hexamers, see discussion below), CcmO pseudo-hexamers (1 copy), and RbcX dimers (3 copies). Additionally, 6 copies of CcmL pentamers were detected in the β-carboxysome, which is consistent with previously reported observations (Sun et al. 2019). Overall, the Syn7942 β-carboxysome had a molecular mass of ∼529 MDa (Table 1).

In contrast to the low copy numbers of β-CA, Rubisco and CcmK2/K3/K4 hexamers accounted for ∼68% and ∼25% of the total molecular mass, respectively. The Rubisco-scaffolding protein CcmM has 2 isoforms: a 35-kDa truncated CcmM35 and a full-length 58-kDa CcmM58 (Long et al. 2007; Cot et al. 2008; Long et al. 2010, 2011). CcmM35 contains 3 SSUL domains that interact with Rubisco (Hagen et al. 2018; Wang et al. 2019), whereas CcmM58 has an N-terminal γ-CA-like domain that has been suggested to interact with β-CA (Pena et al. 2010; Zang et al. 2021), in addition to 3 C-terminal SSUL domains. Both CcmM35 and CcmM58 accounted for 6% of the total molecular mass. Interestingly, the shell–cargo complex linker protein, CcmN, exhibited a very low abundance, accounting for only 0.02% of the total molecular mass, and was not present in a specific stoichiometric ratio to CcmM58.

## Discussion

Carboxysomes are natural organelle-like microcompartments responsible for CO_2_ fixation in cyanobacteria and some proteobacteria, which serve as important players in the global carbon cycle. Due to their self-assembly, encapsulation, and selective permeability properties that facilitate efficient CO_2_ fixation, carboxysomes have attracted considerable interest in basic research and bioengineering applications. In this study, we performed cryoET analysis and absolute quantification using QconCAT-based LC-MS to determine Rubisco packaging and stoichiometric composition of functional, intact Syn7942 β-carboxysomes, a model system widely used in carboxysome studies.

**Figure 5.**
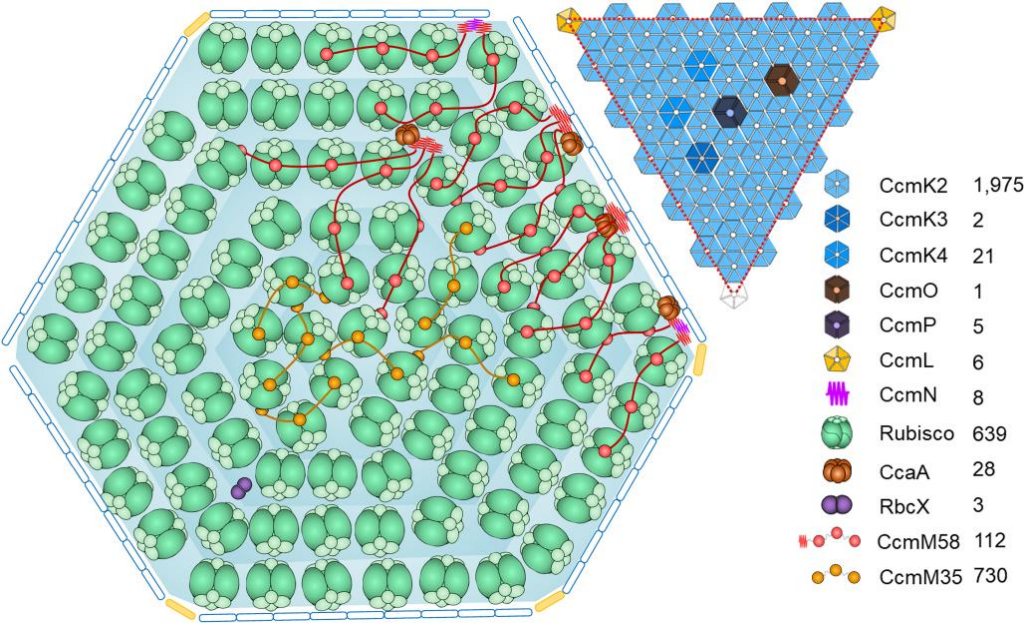
Schematic model of the Syn7942 β-carboxysome structure. The model illustrates the interior organization and shell facet composition determined by QconCAT-determined stoichiometry and cryoET. Rubiscos (green) are organized in multiple layers in the carboxysome. CcmN (fuchsia) and the N-terminus of CcmM58 (red fragment) mediate the Rubisco-shell binding. Rubiscos are crosslinked by SSUL of CcmM58 (red) and CcmM35 (orange). CcaA (brown) and RbcX (purple) are co-encapsulated with the Rubisco matrix. On the shell, CcmK3, CcmK4, CcmO, and CcmP are integrated into the facets formed predominantly by CcmK2 hexamers. The vertexes are partially capped by CcmL pentamers. The numbers of proteins present in the model are for illustration purposes only and do not correlate to actual abundances, which are displayed separately on the right.

Based on our findings, we propose a model of the entire β-carboxysome structure from Syn7942 (Fig. 5). CcmK2 hexamers are the dominant components of the β-carboxysome shell. Other shell proteins, including the CcmK4 and CcmK3 hexamers and the CcmO and CcmP pseudo-hexamers, tile the shell facets. On average, only 6 copies of CcmL pentamers were found per β-carboxysome, which is comparable to the examined CcmL content per β-carboxysome using fluorescence imaging quantification (Sun et al. 2019). It is less than both the theoretical estimation of 12 pentagons per canonical icosahedron and the determined number of CsoS4 pentamers (11 copies) per α-carboxysome (Sun et al. 2022). This discrepancy suggests an increased likelihood of incomplete capping at the vertices of β-carboxysomes and likely leads to the greater structural variability of β-carboxysomes compared to that of α-carboxysomes. More than 600 Rubisco L_8_S_8_ hexadecamers were encapsulated within the Syn7942 β-carboxysome with a diameter of 170 nm. Rubiscos are organized in several concentric layers parallel to the outer shell, with Rubisco complexes oriented predominantly in the radial direction (Supplementary Fig. S4). The CcmN–CcmM assemblies play an essential role in bridging the association between the shell and cargo enzymes. Our results suggest that there are, on average, 8 copies of CcmN in each β-carboxysome, in contrast to 112 copies of CcmM58 and 730 copies of CcmM35. This is strikingly distinct from the previously estimated CcmN:CcmM stoichiometry based on in vitro studies (Sun et al. 2021). Moreover, both our findings and previous structural analysis of reconstituted Rubisco–CcmM complexes (Wang et al. 2019) reveal that the SSUL modules of CcmM58 and CcmM35 bind Rubiscos and crosslink neighboring Rubiscos, mediating Rubisco recruitment and packaging within the β-carboxysome. CcmM58 and CcmM35 may crosslink multiple Rubiscos within the same layer or across different layers. Although each Rubisco has 4 CcmM SSUL-binding sites, our data suggest that the SSUL domains have low occupancy on individual Rubiscos (Fig. 2F), which might provide the foundation for the formation of Rubisco condensates and their organizational flexibility. Additionally, there are 28 CcaA enzymes and 3 RbcX proteins encapsulated within the β-carboxysome lumen, which are essential for the assembly and carboxylation activity of Rubiscos.

This specific Rubisco organization identified may provide the foundation for packaging the maximum number of Rubisco complexes into the limited internal space of the β-carboxysome. This dense packing and concentration of Rubisco is thought to enhance carboxylation reactions of each individual β-carboxysome and help the cell make the most of its available space and resources to maximize its CO_2_-fixing capacity. Moreover, our previous studies have revealed a notable size variation of β-carboxysomes in Syn7942 (ranging from 144 to 208 nm) under various environmental conditions, such as light intensity and CO_2_ availability; larger β-carboxysomes can accommodate more Rubisco complexes, with 367 copies of Rubiscos within the β-carboxysome of 144 nm in diameter compared to 1,507 Rubiscos within a 208 nm-diameter β-carboxysome (Sun et al. 2019). The present work and recent studies (Kong et al. 2024) on Syn7942 β-carboxysomes using cryoET further verified this structural variation. Within a β-carboxysome with a diameter of 169 nm, ∼640 Rubisco enzymes were organized into 5 concentric layers (Fig. 2). A larger β-carboxysome with a size of 193 nm can accommodate an additional Rubisco layer arranged close to the shell, due to the 12 nm distances between neighboring Rubiscos (Supplementary Fig. S3), allowing for over 400 additional Rubiscos and more than 1,200 Rubisco enzymes in total encapsulated within the larger β-carboxysome structure (Kong et al. 2024). The carboxysome diameter, as well as the abundance and packing of interior Rubisco, is important factors in determining the overall architecture and physical mechanics of the intact β-carboxysome (Faulkner et al. 2017). These findings underscore the organizational and functional dynamics and flexibility of native β-carboxysomes, a possible mechanism for their adaptation in response to environmental changes. How specific Rubisco packaging is established within the carboxysome and how it determines precisely the carboxysome functionality and the physiological adaptation of cyanobacterial cells in response to environmental changes remain to be explored.

The findings on β-carboxysomes and α-carboxysomes allow extensive comparisons of the structures and assembly mechanisms of the 2 distinct carboxysome linkages, although the smaller Syn7942 β-carboxysomes characterized in this study were still larger than the previously reported α-carboxysomes. Recently studies have suggested that the middle region of CsoS2 plays a crucial role in determining the size of the α-carboxysome (Li et al. 2024; Oltrogge et al. 2024). In contrast, what determines the size of β-carboxysomes remains unclear. Our results imply that the composition of scaffolding proteins may drive variations in carboxysome diameter. The ratio of short to long isoforms of CcmM is nearly 5 times that of CsoS2 (Supplementary Table S4), which presumably favors cargo packaging rather than cargo–shell interactions in the β-carboxysome (Long et al. 2010; Kinney et al. 2012; Wang et al. 2019; Sun et al. 2021; Zang et al. 2021), resulting in the formation of a large cargo core. In contrast, CcmN, the linker protein that binds directly with shell proteins, has a relatively low abundance (Table 1), suggesting that CcmN is unlikely to be the key factor in determining β-carboxysome size. Nevertheless, despite the difference in the size of the 2 types of carboxysomes, the ratios between the shell proteins and cargo enzymes remained consistent (Supplementary Table S4), which may be fundamental for maintaining complete encapsulation and overall polyhedral architecture. Although Rubisco packaging and CcmM binding have been identified, how low-abundance proteins, such as CcmN and β-CA, are integrated within the β-carboxysome remains unclear.

It has been proposed that α- and β-carboxysomes exploit different assembly pathways. *De novo* assembly of β-carboxysomes exploits the “Core first” mode, in which Rubiscos are first condensed, mediated by CcmM, to form a liquid-like Rubisco matrix core, followed by shell encapsulation (Cameron et al. 2013; Chen et al. 2013). In contrast, the construction of the Rubisco core and shell encapsulation during α-carboxysome assembly, mediated by CsoS2, is assumed to occur in a simultaneous and/or separate fashion (Iancu et al. 2010). The cryoET data by us and others revealed that Rubiscos within Syn7942 β-carboxysomes form multiple concentric layers parallel to the shell, which share a similar organizational pattern as Rubiscos in α-carboxysomes from *Cyanobium* and *Prochlorococcus* but are distinct from the Rubisco packaging in *H. neap* α-carboxysomes (Ni et al. 2022a, 2022b; Evans et al. 2023; Kong et al. 2024; Zhou et al. 2024). While the β-carboxysome structures differ in their 3D shape and quantity of concentric layers, our data revealed that there are no significant differences observed in the arrangement and orientation of Rubiscos within each concentric layer among different layers in Syn7942 β-carboxysomes (Fig. 2, Supplementary Fig. S4). In addition, the ratios of Rubisco to scaffolding proteins CcmM/CsoS2 in Syn7942 and *H. neap* carboxysomes remained relatively consistent. Overall, the remarkable structural variations in carboxysomes from different native host organisms highlight their modular features and reprogrammable potential, which may be vital for microorganisms to adapt carboxysome functions and CO_2_ assimilation to specific ecological niches (Sun et al. 2016, 2019; Nguyen et al. 2024). Our results also suggest that evaluating the architecture of mature carboxysomes alone does not provide a definitive way to differentiate between the distinct assembly mechanisms of α- and β-carboxysomes.

In summary, this study highlights the assembly principles and inherent structural variability of β-carboxysomes, which enables a better understanding of their structural plasticity and functional regulation and facilitates the engineering and reprogramming of carboxysomes for new functions.

## Materials and methods

### Bacterial strains, growth conditions, and carboxysome production

*S. elongatus* PCC 7942 (Syn7942) cells were cultivated in BG-11 medium (Rippka et al. 1979) or BG-11 agar plates with TES (*N*-tris(hydroxymethyl)methyl-2-aminoethanesulfonic acid) buffer pH 8.2 (22.9% w/w of C_6_H_15_NO_6_S) and sodium thiosulfate (0.3% w/w of Na_2_S_2_O_3_), solidified on 1.5% agar (w/v). Syn7942 cells were maintained in 5-L Duran flasks under constant low-light illumination (15 μE m^−2^ s^−1^, measured at the culture surface) with aeration and agitation, which enable the production of β-carboxysomes with a smaller diameter (Faulkner et al. 2017; Sun et al. 2019).

### Carboxysome purification from Syn7942

The β-carboxysome purification was performed as previously described with some modifications (Sun et al. 2016; Faulkner et al. 2017). Syn7942 cells from a 16-L culture were harvested at the late-exponential phase when OD_750_ reached 1.5 to 2. The cell pellet was resuspended and incubated in TE buffer (20 mm Tris–HCl pH 8.0, 0.5 mm EDTA) in the presence of 2% cell lytic B (Sigma Aldrich, USA), 1% protease inhibitor cocktails (Thermo Fisher, UK) and 0.5 mg mL^−1^ lysozyme (Sigma Aldrich) for 1 h prior to cell breakage by glass beads beating. Lysates were incubated overnight with 1% Triton X-100 and 0.5% IGPAL CA-630 for membrane solubilization. Lysates were briefly centrifuged at 3,000 × *g* to remove large debris, and the supernatant was centrifuged at 40,000 × *g* for 1 h to obtain crude β-carboxysome enrichment. The crude enrichments were further treated with 1% *n*-dodecyl-β-D-maltoside and 1% DNase for 2 h and then loaded on an identical step sucrose gradient centrifugation used for recombinant β-carboxysomes. Enriched β-carboxysomes were collected from the 50% to 60% sucrose gradient fractions.

### SDS–PAGE and immunoblot analysis

SDS–PAGE was performed following standard procedures. About 5 to 10 *μ*g purified carboxysome proteins were loaded per well on 15% polyacrylamide gels and stained with Coomassie Brilliant Blue G-250 (Thermo Fisher Scientific, UK). The sizes of the protein bands were referenced to the PageRuler Plus Prestained Protein Ladder (Thermo Fisher Scientific). Immunoblot analysis was performed using primary rabbit polyclonal anti-RbcL (1:10,000 dilution, AS03 037, Agrisera), rabbit polyclonal anti-CcmK2 (1:5,000 dilution, PHY5336S, Phytoab), working with secondary antibody anti-rabbit IgG secondary antibody (1:10,000 dilution, Agrisera), and horseradish peroxidase-conjugated goat anti-mouse IgG secondary antibody (1:10,000 dilution, Agrisera). Images were acquired using the Quant LAS 4000 platform (GE Healthcare Life Sciences). Quantification of band intensities was performed using ImageJ software.

### Rubisco activity assays

^14^CO_2_ fixation assay was performed to determine the CO_2_-fixation rate of purified β-carboxysomes, as described previously (Sun et al. 2016). Three independently purified carboxysomes were calibrated to 0.2 mg mL^−1^ and added Rubisco assay buffer (100 mm EPPS, pH 8.0, and 20 mm MgCl_2_) at 0.1 *μ*g, assayed was performed at 30 °C and initiated with the final concentration of 0.5 mm RuBP. The concentration of HCO_3_ was set to 24 mm for all assays in this study.

### Electron microscopy and data analysis

Electron microscopy was performed as previously described (Faulkner et al. 2017; Huang et al. 2020; Chen et al. 2022; Sun et al. 2022). The purified carboxysomes (∼2 mg mL^−1^) were stained with 3% uranyl acetate on carbon grids and then inspected using an FEI (Field Electron and Ion Company) 120 kV Tecnai G2 Spirit BioTWIN TEM equipped with a Gatan Rio 16 Camera. The diameters of carboxysomes were measured using ImageJ as described previously (Faulkner et al. 2017; Sun et al. 2022).

### CryoET sample preparation, data acquisition, and data analysis

To prepare the cryoET grids for β-carboxysomes, 500 *µ*L purified β-carboxysomes in 60% sucrose were diluted to 5 mL with TEMB buffer (10 mM Tris-HCl, pH 8.0, 1 mM EDTA, 10 mM MgCl_2_, 20 mM NaHCO_3_) and concentrated to 100 *µ*L using 100 K centrifugal filters (Amicon Ultra) by centrifugation at 700 × *g* for 10 min to remove sucrose. Then, 100 *µ*L β-carboxysomes were diluted to 5 mL again and concentrated to a final volume of 50 *µ*L (10× concentrated with 0.6% sucrose in TEMB buffer). The concentrated β-carboxysomes were plunge-frozen in ethane onto lacey holy carbon grids (300 mesh, Agar Scientific) using a Leica GP2. The grids were glow-discharged for 45 s before plunge freezing, and gold fiducial beads (7 nm) were mixed with the sample before sample application to the grids. The excess solution was blotted for 3 or 3.5 s with a humidity of 100% and a temperature of 20 °C.

Optimized cryoET grids were loaded onto a Titan Krios microscope (Thermo Fisher Scientific) operated at 300 keV in the Electron Bio-Imaging Centre, Diamond. The dataset was acquired using a Gatan Quantum post-column energy filter (Gatan Inc.) operated in zero-loss mode with a 20-eV slit width, paired with a Gatan K3 direct electron detector, using Thermo Scientific Tomography 5 Software by electron counting in super-resolution mode at a physical pixel size of 1.35 per pixel. The tilt series was collected using a dose-symmetric tilt scheme starting from 0 ° with a 3 ° tilt increment by a group of 3 and an angular range of ±60 °. The accumulated dose of each tilt series was around 120 e^−^/Å^2^ with a defocus range between −2.5 and −5.5 *μ*m. Ten raw frames were saved for each tilt series. The details of the data collection process are presented in Supplementary Table S1.

For cryoET data analysis, the tilt series for β-carboxysomes were aligned with IMOD (v4.9.12) (Kremer et al. 1996) using gold fiducials, with the aid of an in-house on-the-fly processing Python script (https://github.com/ffyr2w/cet_toolbox). The center of each identified gold fiducial was checked manually. STA was performed using emClarity (v1.5.0.4, v1.6.2) (Ni et al. 2022a, 2022b). The Rubisco crystal structure (PDB ID: 5NV3) was converted to a density map at 20 Å resolution with a molmap command in Chimera and then used as the template for particle searching using emClarity. Template matching was performed using 4× binned tomograms with a pixel size of 5.4 Å (hereafter bin4 tomograms). Rubisco coordinates were manually checked using IMOD to remove false positives and individual Rubiscos outside the β-carboxysome. A total of 185 β-carboxysomes were used for STA and alignment. The averaging and alignment were first performed at bin3 with a pixel size of 4.05 Å for 4 cycles, followed by bin2 with a pixel size of 2.70 Å pixel for 8 cycles, and bin1 with a pixel size of 1.35 Å for 4 cycles. The dataset was divided into 2 independent subsets during alignment for gold-standard metrics, and the 2 subsets were combined in the final iteration, resulting in a final resolution of 3.7 Å. D4 symmetry was applied throughout the alignment procedure and the final density map was reconstructed using 2D tilt series images with cisTEM within the emClarity package at a resolution of 3.5 Å. As for the radial and angular distribution of Rubiscos, the core of each β-carboxysomes was defined as the average of all Rubiscos positions within the β-carboxysome. The distance between each refined Rubisco and the β-carboxysome core was generated to plot the radial distance distribution. A radial vector for each Rubisco was calculated pointing from the core of its corresponding β-carboxysomes to each Rubisco, while the angle between the radial vector and the 4-fold axis or Rubisco was generated to investigate the radial angular distribution.

CryoET of Syn7942 cells was performed as previously described (Huokko et al. 2021). For cell vitrification and cryo-focused ion beam (cryo-FIB) milling, Syn7942 cells were diluted to OD_750_ = ∼0.8 in their culture medium before plunge freezing. Three microliters of suspended cells in a culture medium was applied to the front side of glow-discharged R2/2 carbon-coated copper grids (Quantifoil MicroTools), with additional 1 *µ*L cells added to the backside. The grids were blotted for 4 s before being vitrified in liquid ethane using a Leica GP2 plunge freezer. The vitrified grids were subsequently loaded onto an Aquilos FIB/scanning electron microscope (FIB/SEM) (Thermo Fisher Scientific) to prepare thin lamellae of the cells. To reduce overall specimen charging, the grids were sputter-coated with platinum before milling. The ion beam current was gradually adjusted to lower values as the lamellae thinning progressed (first using 0.1 nA until 0.75 *µ*m thick, and then 50 pA until ∼250 nm thick). For cryoET data collection, tomography tilt series were acquired from lamella using a Titan Krios microscope (Thermo Fisher Scientific) operated at 300 kV. The tilt series data were acquired with a stage pretilt of −7 ° to account for the angle of lamella, making the lamella surface roughly perpendicular to the electron beam. Tilt series were collected from −42 to 42 ° in 2° increment by a group of 3 using a dose-symmetric scheme (Hagen et al. 2017) in SerialEM (Mastronarde 2005), with a target defocus of −10 *µ*m. The tilt series reconstruction was performed with a binning factor of 4, resulting in a final pixel size of 2.492 nm. Micrographs were recorded with a K3 camera equipped with a Gatan Quantum energy filter operated in zero-loss mode with a 20 eV slit width. The exposure time was set to 5 s, and the videos were fractionated into 10 subframes. The nominal magnification of the recorded images was 15,000×, with a pixel size of 6.23 Å. The total accumulated dose was ∼120 e^−^/Å^2^.

### Design, cell-free expression, and purification of QconCAT standard

Absolute quantification of carboxysome protein components was performed using concatenated signature QconCAT peptides (Pratt et al. 2006) in a manner similar to that described previously (Yang et al. 2020; Sun et al. 2022). Briefly, 2 qualified peptide candidates were selected to quantify each protein. Candidate peptides were BLAST searched against the protein database in the Syn7942 database to ensure their uniqueness. The DNA fragment encoding the above peptides, together with GluFib and cMyc, as well as 6× His-tag at the N-terminus and C-terminus, respectively, were generated following the ALACAT/Qbrick assembly strategy, as reported previously (Johnson et al. 2021). The final DNA sequence (Supplementary Table S2) was assembled into a pEU-E01 vector for cell-free expression using wheat germ cell lysates (CellFree Sciences Co. Ltd, Japan). Synthesis was completed with [^13^C_6_, ^15^N_4_] arginine and [^13^C_6_, ^15^N_2_] lysine (CK Isotopes Ltd, UK) using a WEPR8240H full Expression kit following default protocols (2BScientific Ltd, UK). The QconCAT peptides were purified using a HisTrap HP Column (Cytiva, UK) following standard methods. The QconCAT was precipitated and resuspended in 30 *μ*L 25 mm ammonium bicarbonate with 0.1% (w/v) RapiGest SF surfactant (Waters, UK) and protease inhibitors (Roche cOmplete, Mini, EDTA-free Protease Inhibitor Cocktail, Merck, UK).

### Proteomic analysis

Proteomic analysis was performed on 4 independent biological replicates as previously described with modifications (Sun et al. 2022). Designed QconCAT Peptides for β-carboxysome protein quantification were listed in Supplementary Table S2. The overall protein and DNA sequence of QconCAT were provided in Supplementary Table S3. For sample handling, the protein concentration of each sample was determined using a NanoDrop Spectrophotometer (Thermo Fisher Scientific). Carboxysome samples were diluted to a final protein concentration of 2 *μ*g per 72 *μ*L of 50 mm NH_4_HCO_3_. QconCAT (∼0.6 pmol), and the samples were denatured using 8 *μ*L of 10% (w/v) SDS (Thermo Fisher Scientific) in 50 mm NH_4_HCO_3_ followed by incubation at 80 °C for 10 min. Samples were reduced by the addition of 5 *μ*L 12 mm dithiothreitol in 50 mm NH_4_HCO_3_ and incubated at 60 °C for 10 min. Alkylation was carried out by adding 5 *μ*L of 36 mm iodoacetamide in 50 mm NH_4_HCO_3_ and incubation at room temperature for 30 min in the dark. The treated samples were then bound to 40 ng of SP3 Beads (Cytiva, UK) by the addition of acetonitrile to a final concentration of 80% acetonitrile and incubated for 30 min. The beads were washed 3 times with 100% acetonitrile and once with 100% water. The beads were resuspended in 50 mm NH_4_HCO_3_ and the sample was digested with trypsin (1 *μ*L of 200 ng in 50 mm NH_4_HCO_3_ in a final volume of 20 *μ*L). Samples were incubated at 37 °C overnight, followed by centrifugation at 17,200 × *g* for 30 min, and transferred to fresh low-binding tubes. Three biological replicates were analyzed using an UltiMate 3000 RSLCnano system coupled to a Q Exactive HF Hybrid Quadrupole-Orbitrap Mass Spectrometer (Thermo Fisher Scientific) in data-dependent acquisition mode, according to a previously published protocol (Johnson et al. 2021). The LC was operated under the control of Dionex Chromatography MS Link 2.14. The raw MS data files were loaded into Thermo Proteome Discoverer v.2.4 (Thermo Fisher Scientific) and searched against the carboxysome QconCAT database using Mascot v.2.8 (Matrix Science London, UK) with trypsin as the specified enzyme. Each precursor ion was cleanly isolated at the high-resolution scanning speed of the MS1 approach. A precursor mass tolerance of 10 ppm and a fragment ion mass tolerance of 0.01 Da were applied. Data analysis, including run alignment and peak picking, was carried out using Skyline v23.1 (MacCoss Lab Software) (MacLean et al. 2010; Pino et al. 2020). Single carboxysome quantitative normalization was performed using Rubisco counts measured from cryoET, as shown in Fig. 2C.

### Statistical analyses

Data are presented as means and Sd determined from multiple independent biological replicates as specified by *n* for individual experiments. Statistical analysis was conducted using Origin (OriginLab, Massachusetts, USA); statistical significance was determined by Student's *t*-test.

### Accession numbers

Sequence data from this article can be found in the GenBank/EMBL data libraries under accession numbers AAA27304.1 (CcmK2), ABB56316.1 (CcmK3), ABB56317.1 (CcmK4), AAA27305.1 (CcmL), AAA27306.1 (CcmM), AAA27326.1 (CcmN), AAA27327.1 (CcmO), ABB56552.1 (CcmP), ABB57456.1 (RbcL), ABB57457.1 (RbcS), ABB57565.1 (RbcX), and AAA27315.1 (CcaA).

## Supplementary Material

kiae665_Supplementary_Data

## Data Availability

All data needed to evaluate the conclusions are presented in the paper and/or in Supplementary information. The cryoET subtomogram averaging density maps and corresponding atomic models were deposited in the PDB and EMDB, with accession codes 9FWV (https://doi.org/10.2210/pdb9FWV/pdb) and EMD-50836 (https://www.ebi.ac.uk/emdb/EMD-50836), respectively.

## Supplementary data

The following materials are available in the online version of this article.

**Supplementary Figure S1.** Isolation and identification of Syn7942 β-carboxysomes.

**Supplementary Figure S2.** Positioning of Rubiscos in a β-carboxysome by template matching using emClarity.

**Supplementary Figure S3.** Layer profiles of Rubiscos within β-carboxysomes.

**Supplementary Figure S4.** Orientation profile of Rubiscos within β-carboxysomes.

**Supplementary Figure S5.** The density and fittings of the Rubisco small subunit-like (SSUL) domain of CcmM on Rubisco.

**Supplementary Figure S6.** Design and preparation of Quantification conCATamer (QconCAT) quantification.

**Supplementary Table S1.** CryoET data collection, processing and model refinement statistics.

**Supplementary Table S2.** QconCAT peptides derived from tryptic proteolysis for β-carboxysome protein quantification.

**Supplementary Table S3.** DNA and protein sequences of the β-carboxysome QconCAT peptide.

**Supplementary Table S4.** Comparison of the protein ratios of β-carboxysomes from Syn7942 and α-carboxysomes from *H. neapolitanus*.

**Supplementary Video S1.** Template matching results show the Rubisco packaging and the spatial positions of matched Rubiscos within the β-carboxysome.

**Supplementary Video S2.** Structure of Rubisco from β-carboxysomes by cryoET subtomogram averaging.

**Supplementary Video S3.** Binding of CcmM SSUL to Rubisco within the β-carboxysome determined by cryoET subtomogram averaging.

**Supplementary Video S4.** Tomographic slices view of Syn7942 cell lamella.

**Supplementary Data Set 1.** QconCAT data of β-carboxysome proteins within the isolated Syn7942.

## Dive Curated Terms

The following phenotypic, genotypic, and functional terms are of significance to the work described in this paper:

LAS Gramene: AT1G55580
LAS Araport: AT1G55580

electron CHEBI: CHEBI:10545

EDTA Gramene: Ethylene Diamine Tetraacetic Acid
EDTA Araport: Ethylene Diamine Tetraacetic Acid
