# Supplementary material for: Rubisco packaging and stoichiometric composition of the native β-carboxysome in Synechococcus elongatus PCC7942
Source: Plant Physiol. 2024 Dec 16;197(1):kiae665. doi: 10.1093/plphys/kiae665 (PMC11973430; doi:10.1093/plphys/kiae665)
Supplement: kiae665_Supplementary_Data [file kiae665_supplementary_data.zip › suppvideolegends.docx]

**Supplementary Video S1. Template matching results show the Rubisco packaging and the**

**spatial positions of matched Rubiscos within the β-carboxysome.** Individual Rubiscos are

illustrated as green ring-like structures.

**Supplementary Video S2. Structure of Rubisco from β-carboxysomes by cryoET subtomogram**

**averaging.** Slice view from the top to bottom of Rubisco. The structures of Rubisco subunits (RbcL

and RbcS) are coloured blue and orange, respectively.

**Supplementary Video S3. Binding of CcmM-SSUL to Rubisco within the β-carboxysome**

**determined by cryoET subtomogram averaging.** Slice view from the top to bottom of Rubisco.

The structures of Rubisco subunits (RbcL and RbcS) are colored in blue and orange, respectively.

CcmM SSUL proteins in one binding mode (upper groove) are shown in magenta.

**Supplementary Video S4. Tomographic slices view of Syn7942 cell lamella.**
