# Supplementary material for: Rubisco packaging and stoichiometric composition of the native β-carboxysome in Synechococcus elongatus PCC7942
Source: Plant Physiol. 2024 Dec 16;197(1):kiae665. doi: 10.1093/plphys/kiae665 (PMC11973430; doi:10.1093/plphys/kiae665)
Supplement: kiae665_Supplementary_Data [file kiae665_supplementary_data.zip › PP2024RA02517R1_Supplemental_Figures_and_Tables.pdf]

Supplementary Information

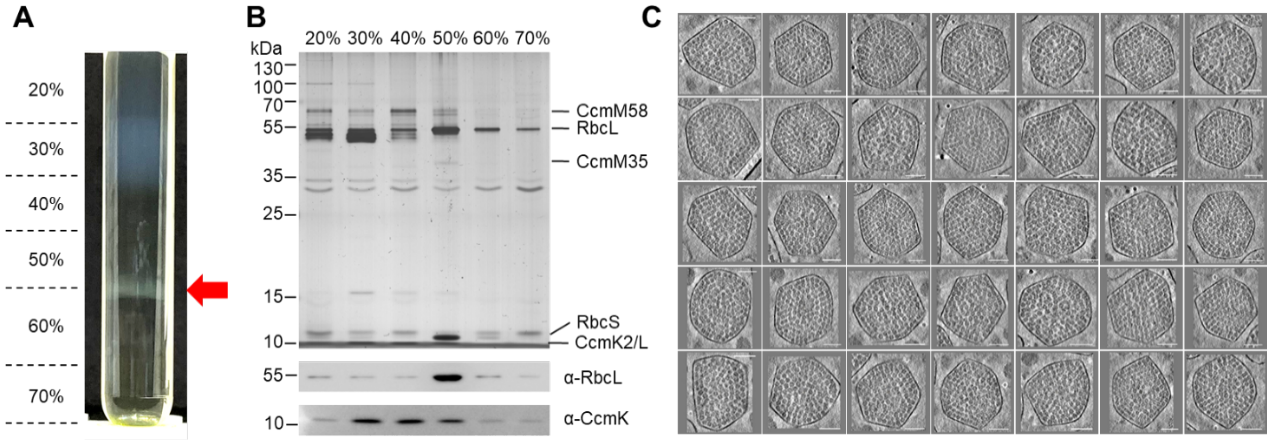

Supplementary Figure 1. Isolation and identification of Syn7942 β-carboxysomes. (**A**) Sucrose gradient purification of β-carboxysomes from Syn7942, the red arrow indicates enriched carboxysomes; (**B**) SDS-PAGE and immunoblots identification for Syn7942 β-carboxysomes in sucrose gradient fractions. The major enzyme Rubisco and main shell CcmK2 were detected by RbcL and CcmK2 antibodies respectively. Intact β-carboxysomes are enriched at the bottom of the 50% sucrose gradient; (**C**) Morphological variations of Syn7942 β-carboxysomes. Scale bar: 50 nm.

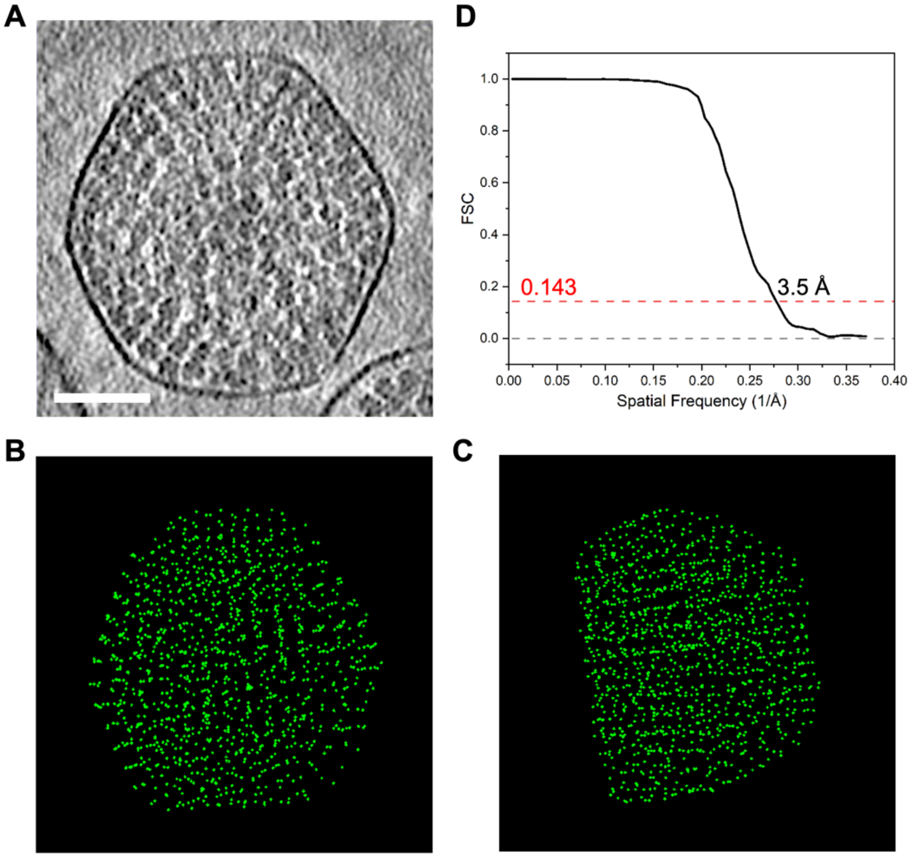

Supplementary Figure 2. Positioning of Rubiscos in a β-carboxysome by template matching using emClarity. (**A**) A tomographic slice of a β-carboxysome. Scale bar: 50 nm. (**B**) Top and (**C**) side projection views of template-matched Rubisco points, indicating ordered arrays of Rubiscos within the β-carboxysome. (**D**) Fourier Shell Correlation (FSC) of Rubiscos from β-carboxysomes. The resolution is 3.5 Å.at FSC of 0.143.

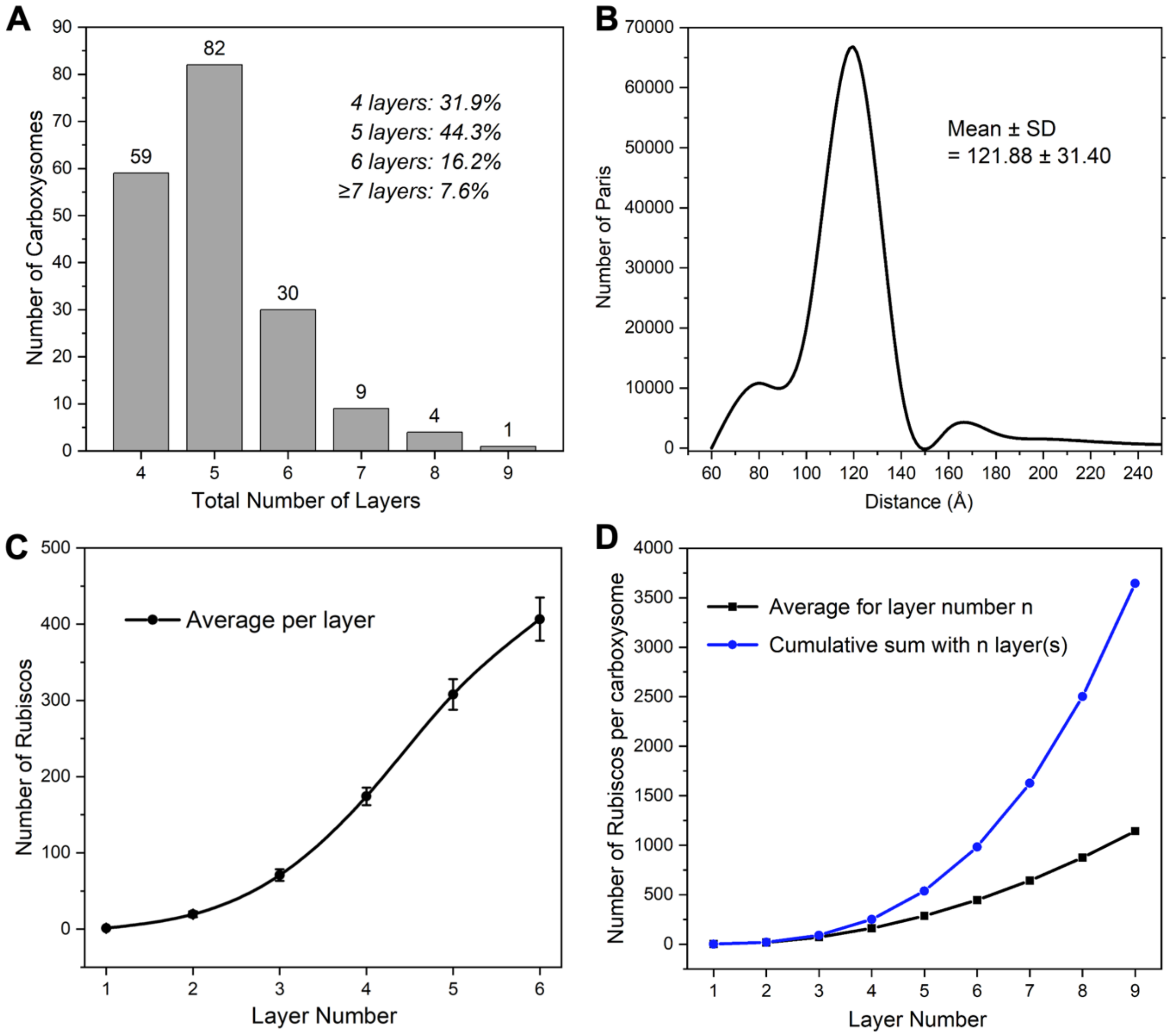

Supplementary Figure 3. Layer profiles of Rubiscos within β-carboxysomes. (**A**) Histogram of layer numbers in β-carboxysomes (*n* = 185). (**B**) Pairwise Rubisco distances in β-carboxysomes (*n*, Rubisco counts = 118k). Distances within 250 Å were plotted. The average distance is 121.88 Å with a standard deviation of 31.40 Å. (**C**) Average number of Rubiscos in a β-carboxysome from layer 1 to layer 6 (*n* = 6 carboxysome particles). (**D**) The predicted number of Rubiscos per β-carboxysome for layer number n (black) and the cumulative sum of Rubiscos in a β-carboxysome with *n* layers (blue) (*n* = 1-9).

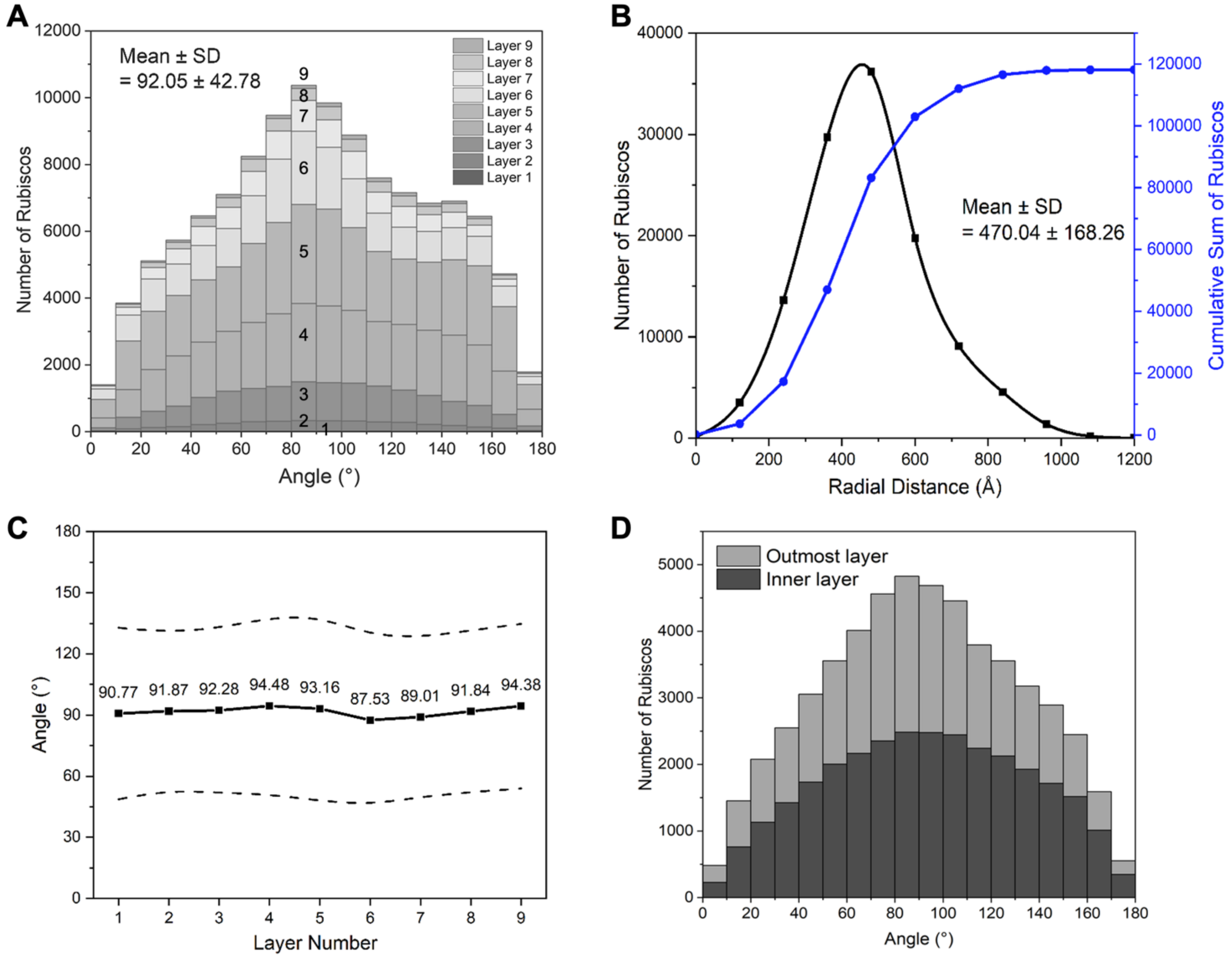

Supplementary Figure 4. Orientation profile of Rubiscos within β-carboxysomes. (**A-B**) Distributions of angle from radial direction (A) and radial distance (B) for Rubiscos in β-carboxysomes (*n* = 118k). (**C**) The average angle (black solid line) of Rubiscos in β-carboxysomes for each layer. The black dashed lines indicate the standard deviation of each layer (*n* = 118k). (**D**) Rubisco angle distribution comparing the outer and inner layers. Angle distribution of Rubiscos in the inner layer is shown in black and the outmost layer shown in light grey. The inner layer contains all the Rubiscos within a radial distance of 360 Å from the core, while the outmost layer has the farthest 20% Rubiscos in each β-carboxysome. The angle for the inner layer is 88.8º on average with a standard deviation of 39.7º, and the average for the outmost layer is 92.9º with a standard deviation of 41.0º.

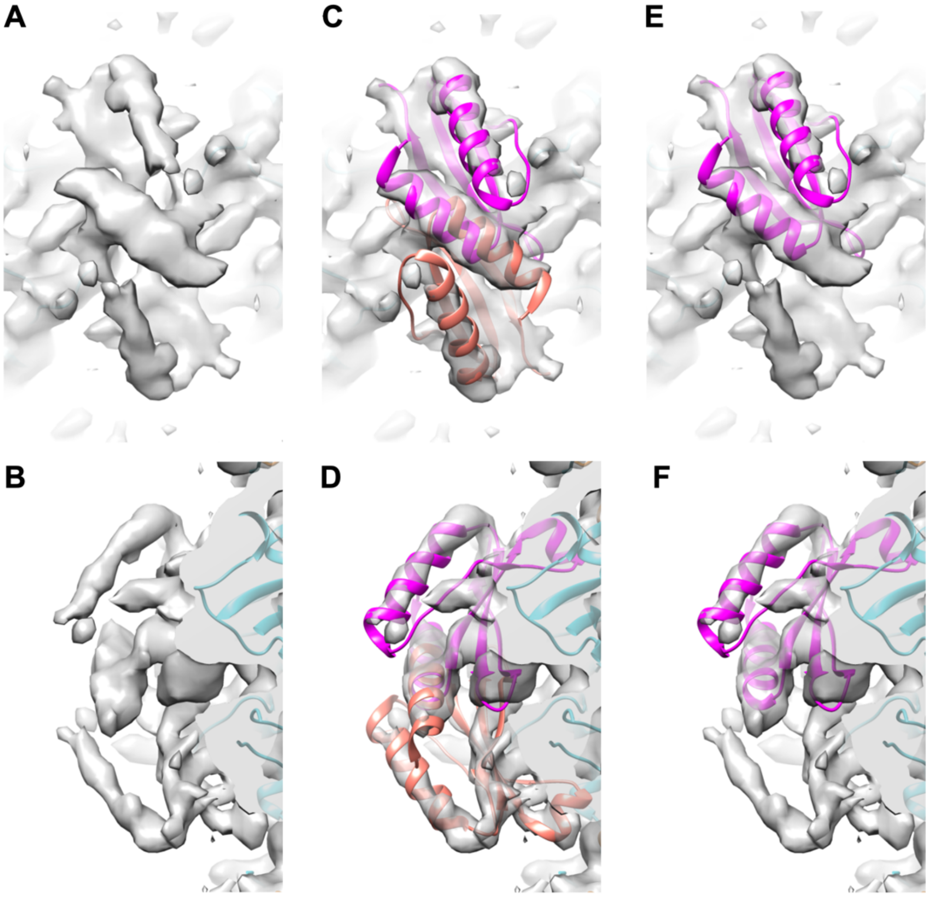

Supplementary Figure 5. The density and fittings of the Rubisco small subunit-like (SSUL) domain of CcmM on Rubisco. (A-B) The top and side views of the extra density. **(C-D)** The top and side views of the extra density, fitted with atomic model of the CcmM SSUL domain (PDB: 6HBC, magenta) to the top density. **(E-F)** The top and side views of the extra density, fitted with atomic model CcmM SSUL (PDB: 6HBC, orange) to the bottom density. D4 symmetry was applied when processing the dataset.

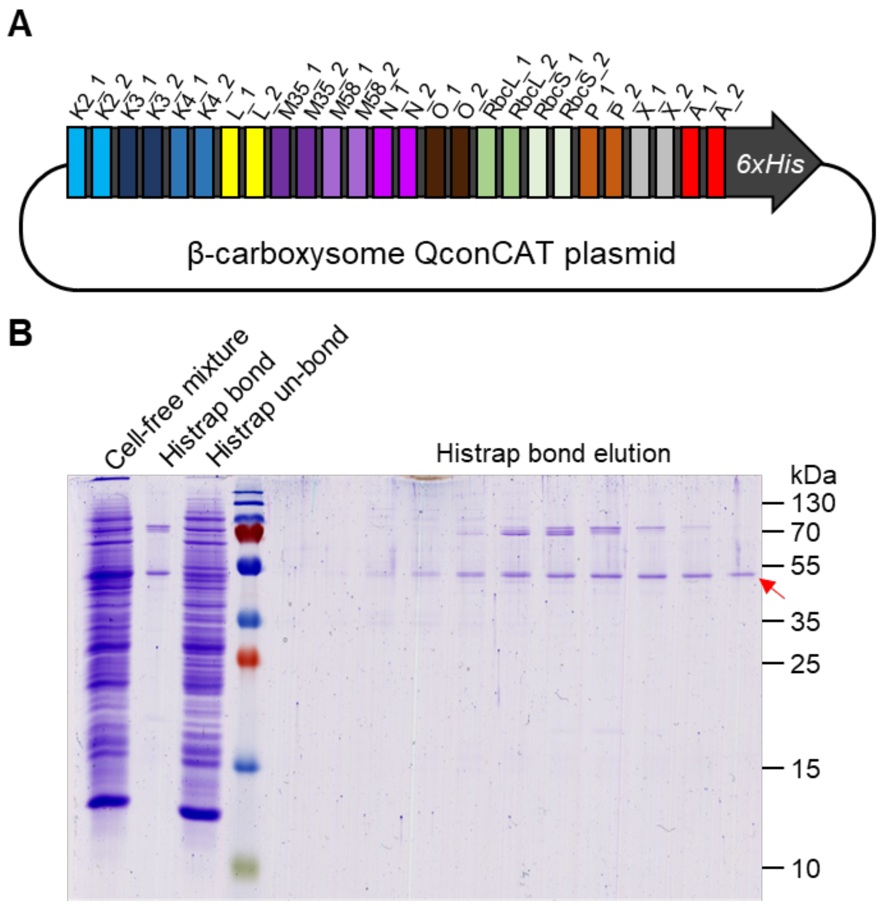

Supplementary Figure 6. Design and preparation of Quantification conCATamer (QconCAT) quantification. (**A**) Design of QconCAT standard peptides, two representative peptides are selected to quantify each β-carboxysome protein. The QconCAT is also His-tagged on the C-terminus, Sequence for each peptide, full DNA and protein sequences are provided in Supplementary Tables 2 and 3; (**B**) SDS-PAGE for cell-free reaction mixture expression QconCAT peptides. The QconCAT standard at a size of 53.7 kDa was synthesized in a cell-free system and purified via the His-trap affinity column. Pure QconCAT standard was obtained in the last fraction of elution, marked by a red arrow.

Supplementary Table 1. Cryo-ET Data collection, processing and model refinement statistics.

Rubisco in the Syn7942 β-carboxysome, EMD-50836, PDB 9FWV	
**Data collection**	
Microscope	FEI Titan Krios
Magnification	64k
Voltage	300 kV
Electron dose	120 $e^{-}/Å^{2}$
Detector	Gatan K3
Energy filter (slit)	Yes, 20 eV
Acquisition scheme	−60°/+60°, 3° dose-symmetric
Frame number	10
Defocus Range	−2.5 to −5.5 μm
Pixel Size	1.35 Å
**Data processing**	
Symmetry imposed	D4
Number of Tilt-series	65
Number of carboxysomes	185
Final particle numbers	87,065
Map resolution(Å)	3.5
FSC threshold	0.143
**Refinement**	
Initial Model used (PDB code)	8BCM, 6HBC
Model composition	
Non-hydrogen atoms	39484
Residues	4952
RMSD	
Bond length(Å)	0.004
Bond angles(°)	0.629
MolProbity score	2.1
Clashscore	16.41
Rotamers Outliers (%)	0.07
Ramachandran Plot	
Favored(%)	94.4
Allowed(%)	5.44
Outliers(%)	0.16

Supplementary Table 2. QconCAT Peptides derived from tryptic proteolysis for β-carboxysome protein quantification. The flanking sequences that recapitulate the true native primary sequence context, together with additional sequences (−3 and +3 seq) which are derived from the loop assembly synthesis of the QconCAT.

Protein	Uniprot	Cyanobase	Candidate	−3 seq	Peptide sequence	+ 3 seq	Full sequence
**CcmK2**	CCMK_SYNE7	Synpcc7942_1421	1	AAR	VTLVGYEK	IGS	AARVTLVGYEKIGS
**CcmK2**	CCMK_SYNE7	Synpcc7942_1421	2	SGR	VTVIVR	GDV	SGRVTVIVRGDV
**CcmK3**	Q31RK3_SYNE7	Synpcc7942_0284	1	SVR	GPVSEVETAVEAGLK	AVA	SVRGPVSEVETAVEAGLKAVA
**CcmK3**	Q31RK3_SYNE7	Synpcc7942_0284	2	AAR	VTITQYGLAESAQFFVSVR	GPV	AARVTITQYGLAESAQFFVSVRGPV
**CcmK4**	Q31RK2_SYNE7	Synpcc7942_0285	1	SAR	GFPPILAAADAMVK	AGR	SARGFPPILAAADAMVKAGR
**CcmK4**	Q31RK2_SYNE7	Synpcc7942_0285	2	SAR	FAVNIR	GDV	SARFAVNIRGDV
**CcmL**	CCML_SYNE7	Synpcc7942_1422	1	KVR	GTVVSTYK	EPS	KVRGTVVSTYKEPS
**CcmL**	CCML_SYNE7	Synpcc7942_1422	2	TYK	EPSLQGVK	FLV	TYKEPSLQGVKFLV
**CcmM35**	CCMM_SYNE7	Synpcc7942_1423	1	RFR	TSSWQSCAPIQSSNER	QVL	RFRTSSWQSCAPIQSSNERQVL
**CcmM35**	CCMM_SYNE7	Synpcc7942_1423	2	QVR	SLLNQGYR	IGT	QVRSLLNQGYRIGT
**CcmM58**	CCMM_SYNE7	Synpcc7942_1423	1	THK	ALIHGPAYLGDDCFVGFR	STV	THKALIHGPAYLGDDCFVGFRSTV
**CcmM58**	CCMM_SYNE7	Synpcc7942_1423	2	PGR	YVPSGAIITTQQQADR	LPE	PGRYVPSGAIITTQQQADRLPE
**CcmN**	Y1424_SYNE7	Synpcc7942_1424	1	DSR	IEIASGVCIGLGSVIHAR	GGA	DSRIEIASGVCIGLGSVIHARGGA
**CcmN**	Y1424_SYNE7	Synpcc7942_1424	2	AGR	SPQSSAIAHPTK	VYG	AGRSPQSSAIAHPTKVYG
**CcmO**	CCMO_SYNE7	Synpcc7942_1425	1	MLK	SADVTLIGYEK	TGS	MLKSADVTLIGYEKTGS
**CcmO**	CCMO_SYNE7	Synpcc7942_1425	2	YEK	TGSGFCTAIIR	GGY	YEKTGSGFCTAIIRGGY
**RbcL**	RBL_SYNE7	Synpcc7942_1426	1	FTK	DDENINSQPFQR	WRD	FTKDDENINSQPFQRWRD
**RbcL**	RBL_SYNE7	Synpcc7942_1426	2	WCR	DNGVLLHIHR	AMH	WCRDNGVLLHIHRAMH
**RbcS**	Q31NB2_SYNE7	Synpcc7942_1427	1	DCK	SPQQVLDEVR	ECR	DCKSPQQVLDEVRECR
**RbcS**	Q31NB2_SYNE7	Synpcc7942_1427	2	MWK	LPLFDCK	SPQ	MWKLPLFDCKSPQ
**CcmP**	Q31QW7_SYNE7	Synpcc7942_0520	1	AEK	AALINILQVSAIGSFGR	LFL	AEKAALINILQVSAIGSFGRLFL
**CcmP**	Q31QW7_SYNE7	Synpcc7942_0520	2	ALK	AAVVRPGVQFIER	LYG	ALKAAVVRPGVQFIERLYG
**RbcX**	Q31N04_SYNE7	Synpcc7942_1535	1	VIR	DQLAETNPAGAYR	LQV	VIRDQLAETNPAGAYRLQV
**RbcX**	Q31N04_SYNE7	Synpcc7942_1535	2	GLR	VMTVR	EHL	GLRVMTVREHL
**CcaA**	CYNT_SYNE7	Synpcc7942_1447	1	ISR	TSSDDTGIDECPVR	LPG	ISRTSSDDTGIDECPVRLPG
**CcaA**	CYNT_SYNE7	Synpcc7942_1447	2	MRK	LIEGLR	HFR	MRKLIEGLRHFR

Supplementary Table 3. DNA and protein sequences of the β-carboxysome QconCAT peptide.

Protein sequence (491 aa)	MGTRMGTREGVNDNEEGFFSARAARVTLVGYEKIGSSGRVTVIVRGDVSVRGPVSEVETAVE<br>AGLKAVAAARVTITQYGLAESAQFFVSVRGPVSARGFPPILAAADAMVKAGRSARFAVNIRG<br>DVKVRGTVVSTYKEPSTYKEPSLQGVKFLVRFRTSSWQSCAPIQSSNERQVLQVRSLLNQGY<br>RIGTTHKALIHGPAYLGDDCFVGFRSTVPGRYVPSGAIITTQQQADRLPEDSRIEIASGVCI<br>GLGSVIHARGGAAGRSPQSSAIAHPTKVYGMLKSADVTLIGYEKTGSYEKTGSGFCTAIIRG<br>GYFTKDDENINSQPFQRWRDWCRDNGVLLHIHRAMHDCKSPQQVLDEVRECRMWKLPLFDCK<br>SPQAEKAALINILQVSAIGSFGRLFLALKAAVVRPGVQFIERLYGVIRDQLAETNPAGAYRL<br>QVGLRVMTVREHLISRTSSDDTGIDECPVRLPGMRKLIEGLRHFRLAAALEHHHHHH
DNA sequence (1,476 bp)	ATGGGTACACGTATGGGCACCCGTGAAGGTGTTAATGATAATGAAGAAGGCTTTTTTAGCGC<br>ACGTGCAGCCCGTGTTACCCTGGTTGGTTATGAAAAAATTGGTAGCAGCGGTCGTGTTACCG<br>TTATTGTTCGTGGTGATGTTAGCGTTCGTGGTCCGGTTAGCGAAGTTGAAACCGCAGTTGAA<br>GCAGGTCTGAAAGCAGTTGCAGCCGCACGTGTTACCATTACACAGTATGGTCTGGCAGAAAG<br>CGCACAGTTTTTTGTTTCAGTGCGTGGTCCTGTTTCAGCCCGTGGTTTTCCGCCTATTCTGG<br>CAGCAGCAGATGCAATGGTTAAAGCAGGTCGTAGTGCACGTTTTGCAGTTAATATTCGCGGT<br>GATGTGAAAGTTCGTGGCACCGTTGTTAGCACCTATAAAGAACCGAGTACATACAAAGAACC<br>GTCACTGCAGGGTGTTAAATTTCTGGTTCGTTTTCGTACCAGCAGCTGGCAGAGCTGTGCAC<br>CGATTCAGAGCAGCAATGAACGTCAGGTTCTGCAGGTTCGTAGCCTGCTGAATCAGGGTTAT<br>CGTATTGGTACAACCCATAAAGCACTGATTCATGGTCCGGCATATCTGGGTGATGATTGTTT<br>TGTTGGTTTTCGTAGTACCGTTCCGGGTCGTTATGTTCCGAGCGGTGCAATTATTACCACAC<br>AGCAGCAGGCCGATCGTCTGCCGGAAGATAGCCGTATTGAAATTGCAAGCGGTGTTTGTATT<br>GGTCTGGGTAGCGTTATTCATGCACGTGGTGGTGCAGCCGGTCGTAGTCCGCAGAGCAGCGC<br>AATTGCACATCCGACCAAAGTTTATGGTATGCTGAAAAGCGCAGATGTGACCCTGATCGGCT<br>ATGAGAAAACCGGTAGTTACGAAAAAACCGGCAGCGGTTTTTGTACCGCAATTATTCGTGGT<br>GGCTATTTCACCAAAGATGACGAAAACATTAATAGCCAGCCGTTTCAGCGTTGGCGTGATTG<br>GTGTCGTGATAATGGTGTTCTGCTGCATATTCATCGTGCAATGCATGATTGTAAAAGTCCGC<br>AGCAGGTTCTGGATGAAGTTCGTGAATGTCGTATGTGGAAACTGCCGCTGTTTGATTGCAAA<br>TCACCGCAGGCAGAAAAAGCAGCCCTGATTAATATCCTGCAGGTTAGCGCCATTGGTAGCTT<br>TGGTCGTCTGTTTCTGGCACTGAAAGCCGCAGTGGTTCGTCCGGGTGTTCAGTTTATTGAAC<br>GTCTGTATGGTGTTATTCGTGATCAGCTGGCCGAAACCAATCCGGCAGGCGCATATCGTCTG<br>CAGGTAGGTCTGCGTGTTATGACCGTTCGTGAACATCTGATTAGCCGTACCAGCTCAGATGA<br>TACCGGCATTGATGAATGTCCGGTTCGTCTGCCTGGTATGCGTAAACTGATTGAAGGTCTGC<br>GCCATTTTCGTCTGGCAGCGGCACTGGAACATCATCACCATCATCATTAG

Supplementary Table 4. Comparison of the protein ratios of β-carboxysomes from Syn7942 and α-carboxysomes from *H. neapolitanus* (Sun *et al*., 2022). *Carbonic anhydrases are considered as the monomeric forms for comparison.

	**Ratio**	
	**Syn7942 β-CB**	***H. neap* α-CB**
Rubisco:scaffolding protein (CcmM or CsoS2)	1:1.2	1:1
Rubisco:carbonic anhydrases*	33:1	31:1
Scaffolding short:long isoforms (CcmM35/58 or CsoS2A/B)	6.3:1	1.3:1
Major shell hexamer:shell-interacting protein (CcmN or CsoS2B)	247:1	5.1:1
Major:minor shell hexamers	86:1	8:1
Shell proteins:cargos	2:1	2:1
Hexamers:trimers	145:1	336:1
Hexamers:pentamers	347:1	134:1
